# Treatment-Resistant Schizophrenia: Insights From Genetic Studies and Machine Learning Approaches

**DOI:** 10.3389/fphar.2019.00617

**Published:** 2019-05-29

**Authors:** Claudia Pisanu, Alessio Squassina

**Affiliations:** ^1^Department of Biomedical Sciences, Section of Neuroscience and Clinical Pharmacology, University of Cagliari, Cagliari, Italy; ^2^Department of Neuroscience, Unit of Functional Pharmacology, Uppsala University, Uppsala, Sweden; ^3^Department of Psychiatry, Dalhousie University, Halifax, NS, Canada

**Keywords:** schizophrenia, antipsychotics, response, clozapine, pharmacogenetics, polygenic risk score

## Abstract

Schizophrenia (SCZ) is a severe psychiatric disorder affecting approximately 23 million people worldwide. It is considered the eighth leading cause of disability according to the World Health Organization and is associated with a significant reduction in life expectancy. Antipsychotics represent the first-choice treatment in SCZ, but approximately 30% of patients fail to respond to acute treatment. These patients are generally defined as treatment-resistant and are eligible for clozapine treatment. Treatment-resistant patients show a more severe course of the disease, but it has been suggested that treatment-resistant schizophrenia (TRS) may constitute a distinct phenotype that is more than just a more severe form of SCZ. TRS is heritable, and genetics has been shown to play an important role in modulating response to antipsychotics. Important efforts have been put into place in order to better understand the genetic architecture of TRS, with the main goal of identifying reliable predictive markers that might improve the management and quality of life of TRS patients. However, the number of candidate gene and genome-wide association studies specifically focused on TRS is limited, and to date, findings do not allow the disentanglement of its polygenic nature. More recent studies implemented polygenic risk score, gene-based and machine learning methods to explore the genetics of TRS, reporting promising findings. In this review, we present an overview on the genetics of TRS, particularly focusing our discussion on studies implementing polygenic approaches.

## Introduction

Schizophrenia (SCZ) is a severe psychiatric disorder that affects approximately 1% of the general population and is associated with a significant socioeconomic burden (Kahn et al., 2015). Antipsychotics represent the mainstay treatment for SCZ, but around one-third of patients show no response (Gillespie et al., 2017). According to the American Psychiatric Association (APA) guidelines, treatment-resistant schizophrenia (TRS) patients are defined as those showing little or no response to at least two non-clozapine antipsychotic trials of adequate duration and dose range (Lehman et al., 2004). Clozapine is the only treatment with an indication for TRS. However, this drug is still underutilized due to monitoring requirement (Kelly et al., 2018) and potential adverse effects, some of which can be severe and life-threatening (Wheeler et al., 2009; Gillespie et al., 2017). Unfavorable response to first-line pharmacological treatments is generally associated with a more severe course of disease in TRS patients (Gillespie et al., 2017; Nucifora et al., 2018). Moreover, TRS patients are highly exposed to the potential detrimental effect of inefficacious treatments, including risk for adverse reactions that could be obviated or reduced if treatment resistance was known in advance.

It has been suggested that differential treatment response to antipsychotics might underlie biologically distinct subphenotypes of SCZ (Farooq et al., 2013; Gillespie et al., 2017) and TRS might better constitute a distinct phenotype rather than just a more severe form of SCZ (Wimberley et al., 2017). In this scenario, it has become clear that TRS patients would significantly benefit from the identification of clinical and biological markers to possibly predict the risk for treatment resistance before starting pharmacological treatments. However, TRS is poorly understood and its neurobiological underpinnings have yet to be clarified.

Data from family studies suggest that TRS is a heritable trait and that heritability might be stronger than in responsive SCZ (Nucifora et al., 2018). Candidate gene and genome-wide association studies (GWAS) have investigated how genetic variation might explain the interindividual variability observed in response to antipsychotics, but only a limited number of them focused on TRS (Nucifora et al., 2018). The majority of these studies used clozapine prescription or treatment as a proxy for diagnosis of TRS, and the investigated genetic variants were selected mainly based on their previously association with SCZ. We can anticipate that findings from these studies have so far not allowed dissecting the genetic complexity underlining TRS. Indeed, it has become clear that TRS is characterized by a complex polygenic nature. Most recent investigations have applied novel approaches such as polygenic risk score (PRS) and kernel support vector machine (SVM) to aggregate the effects of multiple variants contributing to disease risk. These approaches might be better able to capture the polygenic architecture of psychiatric conditions as well as response to psychotropic medications. In light of these observations, in this article, we will review studies investigating the genetic bases of TRS with a focus on studies using polygenic analytical approaches. The articles described in this narrative review were retrieved through a search on PubMed using the following keywords: treatment-resistant schizophrenia, antipsychotic response, genome-wide association study, machine learning, polygenic risk score, and support vector machine.

## Overlap Between Predisposition to SCZ and Response to Antipsychotics

As in the case of other psychiatric disorders, pharmacogenetic studies of antipsychotics have explored the overlap between susceptibility to SCZ and probability to respond to pharmacological treatments. GWASs successfully identified a large number of underlying genetic loci involved in SCZ. Among the most significant efforts, a first mega-analysis of GWAS conducted by the Psychiatric Genomics Consortium (PGC), including 9,394 cases with SCZ and 12,462 controls of European origin, had identified seven genome-wide significant loci (Schizophrenia Psychiatric Genome-Wide Association Study (GWAS) Consortium, 2011). These results were replicated by a GWAS in which patients with SCZ treated with clozapine were compared with healthy controls (Hamshere et al., 2013). More recently, the second mega-analysis of GWAS conducted by the PGC (wave 2), including 36,989 cases and 113,075 controls, identified 108 loci associated with predisposition to SCZ (Schizophrenia Working Group of the Psychiatric Genomics Consortium, 2014). Besides single-nucleotide polymorphisms (SNPs), rare disruptive mutations identified with exome sequencing have also been shown to increase liability for SCZ (Purcell et al., 2014). Based on the evidence that many of the variants identified by genome-wide studies on SCZ are located in genes playing a role in systems likely involved in its neurobiology, there is a rationale for investigating the association between genetic risk for SCZ and response to antipsychotics or TRS. Ruderfer et al. (2016) evaluated both common and rare SCZ-associated loci for enrichment in drug targets, providing interesting evidence that supports the role of some of these genes as drug targets. Authors used a gene-set analysis approach and found that 21% of 167 pharmacological subgroups were enriched for loci previously associated with SCZ. Dopamine receptor D2 (DRD2) was among the loci contributing the most to this finding. Indeed, *DRD2* encodes a known target not only of antipsychotics, but also of 46 different non-antipsychotic pharmacological subgroups out of the 167 evaluated in Ruderfer et al. (2016). The gene set including targets of antipsychotics was enriched for common and rare variants previously associated with SCZ. Authors also compared TRS (532 SCZ patients treated with clozapine) with SCZ patients treated with other antipsychotics (*n* = 2,002), showing a higher number of disruptive mutations in genes targeted by antipsychotics in the TRS group. Taken together, these results support the hypothesis that at least some of the genes identified as involved in the pathogenesis of SCZ might also explain part of the interindividual variability in response to antipsychotics as well as in susceptibility to TRS.

## Genetic Bases of TRS

The number of studies exploring the correlation between genetic variants and TRS is relatively limited. There is still disagreement on the best approach to maximize the power and informativity of genetic studies on TRS, considering the likely complex genetic architecture of this phenotype and the discrepancies on the clinical definition of TRS. The most used operational criteria to define TRS were provided by Kane and colleagues in 1988 (Kane et al., 1988). These criteria include 1) three or more periods of treatment with at least two neuroleptic agents of different classes (1,000 mg/day of chlorpromazine equivalents) for at least 6 weeks, 2) no period of good functioning within the preceding 5 years, and 3) severe psychopathology according to the Brief Psychiatric Rating Scale (BPRS) and Clinical Global Impressions (CGI) scores (Kane et al., 1988). These criteria include more aspects related to functioning compared to the APA guidelines that, as mentioned in the Introduction section, define TRS as little or no response to at least two non-clozapine antipsychotic trials of adequate duration and dose range (Lehman et al., 2004).

Several studies that will be presented in this section used the APA guidelines or a modified version of the criteria defined by Kane et al. (1988), while others used clozapine use or prescription and discontinuation from the prescribed antipsychotic to select TRS patients. It is therefore consequential that the comparison of findings from studies using different definitions may not be straightforward. However, taken together, findings in this field further highlight the importance of the genetic contribution in characterizing TRS.

### Candidate Gene and GWAS

Candidate gene studies investigating the involvement of specific targets in TRS mostly focused on the dopaminergic and serotoninergic systems (Inada et al., 2003; Ji et al., 2008; Kohlrausch et al., 2008; Ota et al., 2012; Bilic et al., 2014; Terzić et al., 2015), as well as on systems involved in inflammation and oxidative stress (Jia et al., 2011; Pinheiro et al., 2017). Among the most interesting findings, Bilic et al. (2014) reported significant interactions between a dopamine transporter variable number tandem repeat (DAT–VNTR) and the serotonin transporter (SERT)-in2 polymorphism in a sample of 172 patients with SCZ, 92 of whom met the TRS definition based on the modified Kane criteria: 1) at least 5 years of inadequate social or occupational functioning, 2) current treatment with a chlorpromazine equivalent dose > 600 mg or score ≥3 on selected items of the Positive and Negative Schizophrenic Symptoms (PANSS) scale or Clinical Global Impression–Severity scale (CGI-S) score ≥4, and 3) history of previous treatment with at least two antipsychotics or history of at least one treatment with clozapine. Results from this study support the hypothesis that, besides the main effect of specific genetic variants, SNP–SNP interactions might also play a role in explaining the interindividual variance observed in response to antipsychotics.

The first large-scale study on the genetic bases underlying response to antipsychotics was conducted in a sample recruited within the Clinical Antipsychotic Trials of Intervention Effectiveness (CATIE) trial (Need et al., 2009). Although this study (which tested 769 polymorphisms in 118 candidate genes) reported several nominal associations, no variant was significantly associated with response to antipsychotics after correction for multiple testing (Need et al., 2009). This study did not specifically include patients with TRS and used discontinuation from the prescribed antipsychotic as a proxy for non-response.

As for large-scale studies, only a small number were conducted in TRS patients. Zhang and coworkers (2013) selected SNPs nominally associated with non-response in the CATIE pharmacogenetic study (Need et al., 2009) and tested their association with TRS (using clozapine treatment as a proxy). Authors reported a significant association between TRS and several genetic variants in linkage disequilibrium (top hit: rs11030104) located in the brain-derived neurotrophic factor (BDNF) gene. Li and Meltzer (2014) conducted a GWAS on two cohorts of Caucasian patients with (*n* = 79 and *n* = 70) and without (*n* = 95 and *n* = 125) TRS. In this study, treatment resistance was defined as persistence of moderate to severe positive symptoms despite at least two trials of 4–6 weeks with typical or atypical antipsychotics other than clozapine. Although no SNP met the genome-wide significant threshold, interesting results were reported for the rs2237457 variant located in 7p12 (*p* value in the combined cohorts: 5.66 × 10^−7^). This variant is located upstream of the gene encoding L-dopa decarboxylase, a rate-limiting enzyme in the synthesis of trace amines and neurotransmitters, including dopamine (Li and Meltzer, 2014). This result is particularly important based on the fact that a hyperdopaminergic state in the mesolimbic dopamine pathway is thought to play a crucial role in the development of psychotic symptoms according to the dopamine hypothesis of SCZ and that dopamine D2 receptors represent a main target for all antipsychotic drugs (Li et al., 2016).

Conversely, Teo and coworkers (2012) did not identify any significant association among 384 candidate gene loci in a study including 85 patients with TRS defined according to APA criteria and 155 non-resistant patients. Other studies focused on the association of genetic variants and clinical factors hypothesized to play a role in the development of TRS, such as childhood adversities. In a GWAS on a sample of 85 Caucasian patients with SCZ [31 of whom met the criteria for TRS defined according to the APA criteria (Lehman et al., 2004)], no SNP met the genome-wide significant threshold for association with TRS with or without taking into account history of childhood adversities (Koga et al., 2017).

### Studies Using PRS

PRS analysis aggregates the effect sizes of several SNPs across the genome, thus providing a single estimate of the association with a specific trait or disease (Dudbridge, 2013). In the last few years, PRS analysis has been successfully applied to the study of different psychiatric disorders (Bipolar Disorder and Schizophrenia Working Group of the Psychiatric Genomics Consortium, 2018; Kalman et al., 2018;Taylor et al., 2018; Richards et al., 2019). In the case of SCZ, PRS analysis has been used to evaluate how the polygenic burden can explain differences in specific symptoms (Wang et al., 2018; Anderson-Schmidt et al., 2019), functional and structural brain changes (Lieslehto et al., 2018; Ranlund et al., 2018; Velthorst et al., 2018), genetic overlap with other traits (International Consortium on Lithium Genetics (ConLi+Gen) et al., 2018), as well as gene co-expression networks in the brain (Radulescu et al., 2018). Studies investigating the potential value of PRS analysis to identify patients that are less likely to respond to treatment provided contrasting findings. In patients with first-episode psychosis (FEP), Zhang and colleagues (2018) found that participants with lower SCZ polygenic burden were more likely to respond to a 12-week antipsychotic treatment compared to patients with high SCZ PRS (odds ratio = 1.91). Conversely, although Santoro et al. (2018) reported a positive association between depressive symptoms and PRS at baseline in a sample of 60 FEP patients, after treatment with risperidone, patients with a higher SCZ PRS were more likely to show improvement in depressive and excitement symptoms. Of note, the study by Santoro and coworkers only included antipsychotic-naive patients.

Studies including patients with TRS also provided controversial findings (**Table 1**). Frank and coworkers (2015) reported that a PRS including SNPs associated with SCZ risk in the first meta-analysis from the PGC Schizophrenia group (Schizophrenia Psychiatric Genome-Wide Association Study (GWAS) Consortium, 2011) was increased in patients with SCZ treated with clozapine compared to patients not treated with this drug. Moreover, the highest PRS was observed in patients who are non-responders to clozapine and characterized by early age at onset and poor premorbid social functioning (Frank et al., 2015).

**Table 1:** Studies investigating the genetic bases of treatment-resistant schizophrenia (TRS) using a polygenic risk score (PRS).

Study	Discovery sample	Target sample	Treatment-resistance criteria	Results
Frank et al. (2015)	First meta-analysis from the PGC Schizophrenia group (Schizophrenia Psychiatric Genome-Wide Association Study Consortium, 2011)	804 German patients with SCZ (434 with TRS)	History of clozapine treatment	Higher PRS in patients treated with clozapine compared to patients with no history of clozapine treatment. The highest PRS was observed in patients characterized by non-response to clozapine, early age at onset and poor premorbid social functioning
Martin and Mowry (2016)	Second meta-analysis from the PGC Schizophrenia group (Schizophrenia Working Group of the Psychiatric Genomics Consortium, 2014)	612 Australian patients with SCZ (227 with TRS)	Poor functioning, continuous course of illness, and at least two among delusions, hallucinations, disorganization and negative symptoms during treatment with antipsychotics	No association between the PRS and non-response to antipsychotics. Association between TRS and total duplication burden genome-wide
Wimberley et al. (2017)	Second meta-analysis from the PGC Schizophrenia group (Schizophrenia Working Group of the Psychiatric Genomics Consortium, 2014), excluding the Danish cases	862 participants with SCZ (181 with TRS) included in the Danish Newborn Screening Biobank	Clozapine initiation or hospitalization during antipsychotic treatment after at least two periods of different antipsychotics monotherapy	No association between the PRS and TRS

PGC, psychiatric genomics consortium; PRS, polygenic risk score; SCZ, schizophrenia; TRS, treatment-resistant schizophrenia.

On the other hand, using the population-based Danish register, Wimberley and coworkers found no association between a PRS for SCZ and TRS (Wimberley et al., 2017). In this study, the PRS was computed based on 24,755 SNPs from the PGC wave 2 paper, using a *p*-value threshold of 0.05 (Schizophrenia Working Group of the Psychiatric Genomics Consortium, 2014), and tested into an independent Danish cohort of 862 patients (181 of whom were considered to be treatment-resistant). In this study, treatment resistance was defined as either 1) clozapine initiation or 2) hospitalization during antipsychotic treatment after at least two periods of different antipsychotics monotherapy (Wimberley et al., 2017). Similarly, Martin and Mowry (2016) found no association between a PRS for SCZ and non-response to antipsychotics in a sample of 612 Australian patients with SCZ (227 of whom showed treatment resistance). In this study, patients were considered to be treatment-resistant in case they showed poor functioning, continuous course of illness, and at least two among delusions, hallucinations, disorganization, and negative symptoms during treatment with antipsychotics. Similarly to other works, the PRS was computed based on SNPs identified by the most recent PGC wave 2 GWAS mega-analysis (Schizophrenia Working Group of the Psychiatric Genomics Consortium, 2014), although a different *p*-value threshold was used (*p* < 0.1). Of note, the authors found a significant association between TRS and the total copy number duplication burden genome-wide (Martin and Mowry, 2016). A number of factors might explain the observed discrepancies between studies investigating genetic bases of TRS using PRS models. For instance, the different *p*-value thresholds chosen by the authors contribute to make these results difficult to compare. Moreover, the sample size of the available studies was generally limited, as the number of subjects with a diagnosis of TRS ranged from 181 to 434 (**Table 1**). To date, available studies only used PRS including SCZ risk variants. However, the development of a PRS specific for TRS might be of high relevance to better capture the contribution of loci underlying non-response to antipsychotics. Moreover, studies evaluating the contribution of a PRS together with clinical characteristics previously identified to be associated with TRS (e.g., earlier age at onset, family history of psychosis and history of substance abuse) would be of high interest.

## Comparison Between PRS and Other Machine Learning Methods

PRS analysis aggregates the contribution of multiple SNPs assuming an additive effect. While this approach has proven to be extremely useful, it doesn’t allow to take into account the potential interactions between different genetic variants. A recent study tried to address this gap using SVM algorithms (Vivian-Griffiths et al., 2019). SVM is a family of supervised learning methods that can be usefully applied to linear as well as non-linear and high-dimensional classification problems (Cai et al., 2001). Specifically, the SVM method allows one to find the optimum hyperplane that separates observations into different classes. Vivian-Griffiths and coworkers (2019) evaluated how this method (allowing to take into account pairwise and higher-order SNP interactions) might help to distinguish patients with TRS from healthy controls compared to a PRS. Models were based on 1) 125 genome-wide significant SNPs and 2) 4,998 independent top SNPs from the PGC wave 2 GWAS mega-analysis (Schizophrenia Working Group of the Psychiatric Genomics Consortium, 2014). The study was conducted in the CLOZUK sample (Hamshere et al., 2013), including 5,554 patients receiving clozapine and 6,299 healthy controls. In this study, two different typologies of SVM models were constructed: SVM with linear and radial basis function kernels. Predictive performances were measured using the area under the receiver operating characteristics curve metric (AUC-ROC) and compared between these two models as well as against those of a PRS. The standardized reference allele counts for each polymorphism were used as input for the SVM model. While no evidence of interaction was found when analyzing the 125 top hits, findings from this study suggested the potential presence of interactions in the models including the more weakly SCZ-associated variants (Vivian-Griffiths et al., 2019). Although the SVM method might be more suitable to take into account these effects, the PRS model showed a higher accuracy in the classification of patients with SCZ treated with clozapine compared with controls. Nonetheless, the prediction accuracy shown by the PRS is still insufficient to support the implementation of the model into the clinical practice [best area under the receiver operating characteristics curve (AUC-ROC) = 0.697].

## Conclusions

A growing number of studies provided intriguing hints on the complex polygenic architecture underlying TRS. However, to date, no genetic marker showed adequate prediction accuracy. Moreover, controversial findings were reported as regards the ability of available PRSs to discriminate individuals with or without TRS. The majority of available studies investigated the effect of variants previously associated with predisposition to SCZ. Indeed, it has been shown that antipsychotics’ targets are enriched for variants previously associated with SCZ (Ruderfer et al., 2016), supporting the need of investigating a potential role for these genes in TRS development. However, in order to better capture the contribution of variants specifically implicated in response to antipsychotics, a more comprehensive approach might involve construction of a PRS specific for TRS (i.e., constructed using genetic data from patients characterized for response to antipsychotics) as such a score might show a higher predictive accuracy. The importance of this aspect is highlighted by the fact that the total duplication burden genome-wide has been associated with TRS (Martin and Mowry, 2016) but not with predisposition to SCZ (Buizer-Voskamp and Muntjewerff, 2011), supporting the hypothesis that only a part of TRS susceptibility might be explained by previously investigated targets.

Another aspect that limits our interpretation of available studies consists in the different criteria used by different researchers to define TRS. While some of the studies adopted the criteria suggested by APA (which define TRS as little or no response to at least two non-clozapine antipsychotic trials of adequate duration and dose range), other studies used different criteria [e.g., modified versions of the Kane criteria (Kane et al., 1988), which take into account different aspects, including global assessment of functioning] or simply considered history of clozapine treatment as a proxy of TRS. Although the latter approach is easier to apply (particularly in the case of studies performed using data from registers) and can therefore lead to studies with an increased sample size, it might lead to an underestimation of the number of patients with TRS. In fact, although clozapine is the only antipsychotic with an indication for TRS, this drug is currently underutilized due to possibly life-threatening adverse reactions as well as to the need of regular monitoring (Remington et al., 2016). In order to minimize this risk, some studies included not only patients treated with clozapine but also participants who would meet the criteria for initiating clozapine treatment based on the data extracted from population-based registers (Wimberley et al., 2017). Nevertheless, the use of standardized criteria to identify patients affected by TRS should be one of the main goals of future efforts, as this would allow evaluating the reproducibility and robustness of findings and the aggregation of available results in meta-analyses, ultimately leading us closer to the understanding of the genetic architecture of TRS.

## Author Contributions

AS and CP searched the literature and compiled the review with equal contribution.

## Funding

CP is supported by Fondazione di Sardegna (“Convenzione triennale 2015–2017”) and by the Autonomous Region of Sardinia (“Legge Regionale 7 agosto 2007, n. 7 – call 2016”), grant number F72F16003090002. Publication fees were covered with funding from a project funded by Regione Atonoma della Sardegna, L.R. 7, call 2013, grant number F72I15000830002.

## Conflict of Interest Statement

The authors declare that the research was conducted in the absence of any commercial or financial relationships that could be construed as a potential conflict of interest.
